# Supplementary material for: Dynamic defect correlations dominate activated electronic transport in SrTiO3
Source: Sci Rep. 2016 Jul 22;6:30141. doi: 10.1038/srep30141 (PMC4957113; doi:10.1038/srep30141)
Supplement: Supplementary Information [file srep30141-s1.pdf]

Supplementary information for "Dynamic defect correlations dominate activated electronic transport in $SrTiO_3$

Paul C. Snijders, Cengiz Şen*, *Michael P. McConnell*, *Ying-Zhong Ma*, *Andrew F. May*, *Andreas Herklotz*, *Anthony T. Wong*, and *T. Zac Ward**

X-ray diffraction

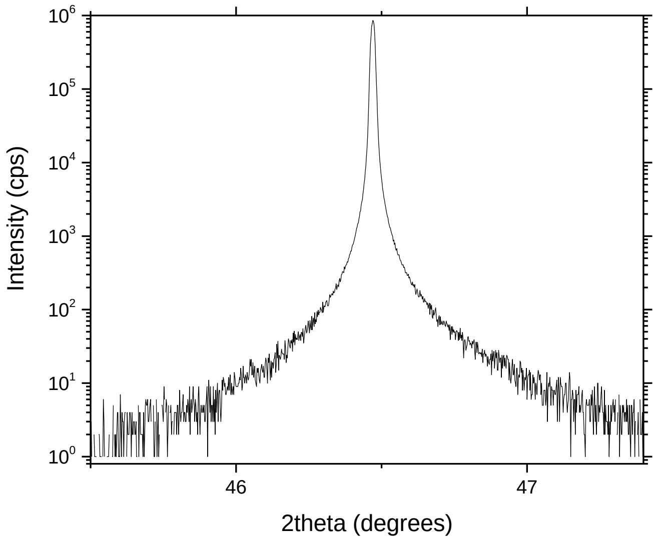

θ-2θ scan of sample C. Oxygen vacancies as well as cation off-stoichiometry[S2] will increase the STO lattice constant above its bulk. Both the out of plane (c) lattice constant of 3.905 Å, i.e. equal to that of relaxed bulk STO, and the absence of shoulders in the (002) reflection show that no defect-induced unit cell volume increases are present within the detection limit of X-ray diffraction, even for this quenched sample C that presumably has the highest defect concentration.

Comparison of experimental relaxation data with results from Density Functional Theory (DFT) calculations.

As discussed in the main text (Table 1), our annealing conditions are expected to create O and Ti vacancies, as well as Sr_{Ti} antisite defects. In an attempt to identify the defects dominating the observed decay in photoconductivity, we compare our experimental data with the results of recent DFT calculations reported in the literature.[S2,S3,S4,S5] While we recognize that this comparison is limited by the small number of defect (complexes) that have been studied using state of the art DFT calculations, and potentially suffers from inaccuracies in DFT band gap determinations, DFT data are the only source of a direct relation between a specific defect type and the energy of its defect state within the STO band gap; experimental methods either average spectroscopic data over many different defects, or introduce the very same uncertainties by the need to be aided by theoretical (DFT) calculations to interpret atomically resolved spectroscopic data.

Oxygen vacancies.

Recent DFT calculations predict[S2] that oxygen vacancies are most stable when doubly ionized and forming a complex with a small polaron. This produces a localized, singly filled defect state located in the gap about 0.4 to 0.5 eV below the conduction band, and puts one electron in the conduction band. Clearly the energetic position of this level, as well as the conductivity that should arise from the induced conduction band carrier density is not observed in our measurements, and hence we believe a model based on independent oxygen vacancies does not describe our results. Another DFT study predicts[S4] that for very high carrier densities $\geq 10^{20}$ cm^{-3}, two electrons can be trapped near an oxygen vacancy by two small polarons. Below this critical

carrier density, the system contains delocalized charge carriers or delocalized large polarons. As our samples were still visually transparent (except for the quench-cooled sample C) and highly resistive, we conclude that we did not create a defect density larger than this critical density. Our samples should therefore conduct reasonably well due to the polaron delocalization at low carrier densities, but instead they are highly resistive, implying that the defect configuration in our samples has to differ from those considered in Ref. [S4].

Ti vacancies.

While there are not many studies considering Ti vacancies, presumably because of their high formation energy,[S2,S5] the formation energy of Ti vacancies should decrease when SrO is present.[S3,S5] DFT calculations show that Ti vacancies are most stable as deep acceptors with a -4 charge state.[S2,S3] Upon photoexcitation with light just below the bandgap, an electron will be excited from this acceptor state into the conduction band. While recombination of the electron in the conduction band with the hole on the defect state does result in a photon emission peak at 1.2 eV because the state of V_{Ti}^{-3} is located 1.2 eV below the conduction band,[S2] there is no energetic barrier for this capture process, which is necessary to explain the observed long term persistence of the photoconductivity - unless the hole on the Ti vacancy recombines with an electron from the valence band, but this corresponds to a transition energy of 2.1 eV, and leaves an excited electron in the conduction band. The latter recombination would not result in the observed decreasing persistent conductivity. As a result, we conclude that isolated Ti vacancies are not likely candidates to explain the observed persistent photoconductivity.

Sr_{Ti} antisite defects.

Finally, a recent comprehensive study on defect complexes in STO[S3] considers the presence of excess SrO, which is comparable to our experimental conditions. It was shown that oxygen vacancies and Sr_{Ti}^{-2} antisite defects are most prevalent under these conditions, but no energetics for in-gap states were provided. However, the generally lower formation energies of the considered defect complexes as compared to isolated point defects, points to the critical importance of such complexes as compared to individual defects in STO.

Supplementary references

[S1] Keeble, D.J., Wicklein, S., Jin, L., Jia, C.L., Egger, W., and Dittmann, R., Nonstoichiometry accommodation in $SrTiO_3$ thin films studied by positron annihilation and electron microscopy. *Phys. Rev. B* **87**, 195409 (2013).

[S2] Janotti, A., Varley, J.B., Choi, M., Van de Walle, C.G. Vacancies and small polarons in $SrTiO_3$. *Phys. Rev. B* **90**, 085202 (2014).

[S3] Liu, B., Cooper, V.R., Xu, H., Xiao, H., Zhang, Y., Weber, W.J. Composition dependent intrinsic defect structures in $SrTiO_3$. *Phys. Chem. Chem. Phys*. **16**, 15590-15596 (2014).

[S4] Hao, X. Wang, Z., Schmid, M., Diebold, U., Franchini, C. Coexistence of trapped and free excess electrons in $SrTiO_3$. *Phys. Rev. B* **91**, 085204 (2015).

[S5] Ohtomo, A. Hwang, H.Y. A high-mobility electron gas at the $LaAlO_3$/$SrTiO_3$ heterointerface. *Nature* ***427***, 423-426 (2004).
